# Potential Effect of Leukocyte-Platelet-Rich Fibrin in Bone Healing of Skull Base: A Pilot Study

**DOI:** 10.1155/2017/1231870

**Published:** 2017-11-16

**Authors:** Felipe Fredes, Jaime Pinto, Nelson Pinto, Pablo Rojas, Daniel M. Prevedello, Ricardo L. Carrau, Thomas Schmidt

**Affiliations:** ^1^Department of Otolaryngology-Head & Neck Surgery, Universidad de Concepción, Concepción, Chile; ^2^Department of Neurosurgery, Universidad de Concepción, Concepción, Chile; ^3^Graduate School of Periodontics and Implant Dentistry, University of the Andes, Research Center for Regenerative Medicine and Tissue Engineering, Concepción, Chile; ^4^Department of Otolaryngology-Head & Neck Surgery, The Ohio State University Wexner Medical Center, Columbus, OH 43210, USA; ^5^Department of Neurologic Surgery, The Ohio State University Wexner Medical Center, Columbus, OH 43210, USA

## Abstract

**Background:**

Reconstruction of surgical defects following cranial base surgery is challenging. Others have demonstrated that leukocyte-platelet-rich fibrin (L-PRF) stimulates tissue healing and bone regeneration. However, these studies have addressed mostly maxillofacial surgical wounds.

**Objective:**

The objective of this study was to assess the possible adjuvant role of L-PRF in inducing neoossification of the surgical bone defect in anterior skull base surgery.

**Methods:**

We identified patients who had undergone an endoscopic endonasal surgery of the anterior skull base in which L-PRF membranes were used for the reconstruction of the bone defect and who were followed up with postoperative CT scans. CT findings were then correlated with baseline scans and with the CT scans of a patient who had undergone imaging and histologic analysis after maxillofacial surgery in which L-PRF was used and in which we demonstrated bone formation.

**Results:**

Five patients fulfilled the inclusion criteria. In four patients, the CT scan demonstrated closure of the bony defect by neoosteogenesis; however, the bone appeared less dense than the surrounding normal bone. A comparison with the control patient yielded similar radiological features.

**Conclusion:**

This case series suggests that L-PRF may induce bone healing and regeneration at the surgical site defect. Multi-institutional studies with a larger series of patients are required to confirm this possibility.

## 1. Introduction

The endonasal endoscopic approach for lesions of the anterior skull base has decreased the morbidity associated with open approaches while achieving similar results in terms of disease control. The use of vascularized tissues to repair surgical defects has dramatically decreased the rate of postoperative complications such as cerebrospinal fluid (CSF) leaks and infections [1]. The introduction of the nasoseptal flap in 2006 reduced the incidence of CSF leaks from 30% to 5% [2]. Despite these advances in reconstructive skull base surgery, complications continue to be reported, so the search for new therapeutic alternatives is necessary to further reduce morbidity.

The use of platelet concentrates in surgery and wound management is an attractive option to enhance tissue healing. One of these products is the leucocyte and platelet-rich fibrin (L-PRF), a platelet concentrate characterized by containing a dense matrix of fibrin, platelets, and leucocytes, thus representing a considerable improvement over its predecessor, platelet-rich plasma (PRP) [3]. PRP products are preparations without leukocytes and with a low-density fibrin network, and all the products of this family can be used as liquid solutions or in an activated gel form, but not as a solid filling material like L-PRF [3]. L-PRF is a simple, natural, and inexpensive product obtained from the patient's blood, which is centrifuged without using anticoagulants. Coagulation occurs during centrifugation, leading to the separation of the blood into three parts: acellular plasma as a supernatant on the top, a fibrin clot rich in platelets and leukocytes in the middle, and red blood cells at the bottom. Compression turns this fibrin clot into a membrane, which can be used to fill surgical defects and/or accelerate healing [4]. It releases growth factors for 7 days, including platelet-derived growth factor (PDGF), transforming growth factor beta 1 (TGFß-1), and vascular endothelial growth factor (VEGF) associated with key coagulation and matrix proteins, such as thrombospondin-1, fibronectin, and vitronectin, thus affording the potential to serve as an adjuvant for stimulating and accelerating healing and/or regenerating tissues [5]. In contrast, PRP products release most of their growth factors in the first hours and completely dissolve after 3 days [3]. Considering the above-mentioned facts, L-PRF has been extensively used in sports and reconstructive medicine, in advanced wound management, and in dental and maxillofacial surgery, with implantology being its main area of development.

In vitro studies showed that L-PRF stimulates cell proliferation and differentiation of mesenchymal stem cells to osteoblasts, promoting new bone formation [6]. This effect seems to be dose-dependent [7]. Studies in the area of implantology have shown the effectiveness of L-PRF in the neoossification of bone at the site of implant placement by imaging and histologic evaluation. While the image of the neoossification area seems less dense than normal bone, histology has confirmed the presence of normal trabecular bone [4, 8]. These findings make L-PRF an interesting option for reconstructive surgery of the anterior skull base by inducing neoossification at the surgical defect.

Therefore, the aim of this pilot study was to assess the possible bone healing by induction of neoossification using L-PRF in patients who underwent endoscopic anterior skull base surgery.

## 2. Methods

This is a descriptive and prospective study of patients who underwent endoscopic skull base surgery with subsequent reconstruction reinforced by L-PRF from 10/2015 to 12/2015.

After a minimum of 3 months after surgery, a high-resolution CT scan was performed to assess the bone growth along the skull base and to compare these images with those of the preoperative CT scans.

**Figure 1:**
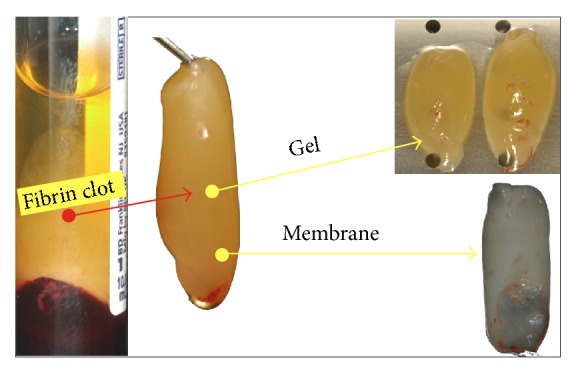
Preparation of L-PRF.

The preparation of L-PRF membranes followed a strict protocol. First, 10 mL of blood per tube was drawn (average 8 to 16 tubes per patient). Within a minute of having been drawn, samples were submitted to centrifugation for 12 to 18 minutes at 2700 rpm (Intraspin L-PRF® centrifuge). Then, the obtained fibrin clot was separated from the acellular plasma (supernatant) and the red blood cells (precipitate), and the fibrin clot was compressed for 3 minutes to obtain L-PRF membranes, with subsequent placement at the intervention site (Figure 1).

**Figure 2:**
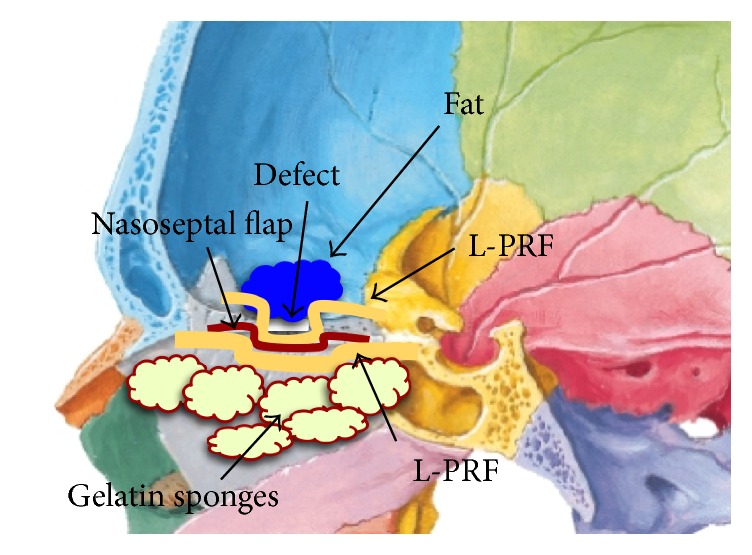
Repair of the surgical defect using L-PRF.

The surgical defect was reconstructed using fat or fascia lata, followed by four to eight L-PRF membranes under the nasoseptal flap. Over the nasoseptal flap, we placed four to eight more L-PRF membranes and gelfoam (Figure 2).

**Figure 3:**
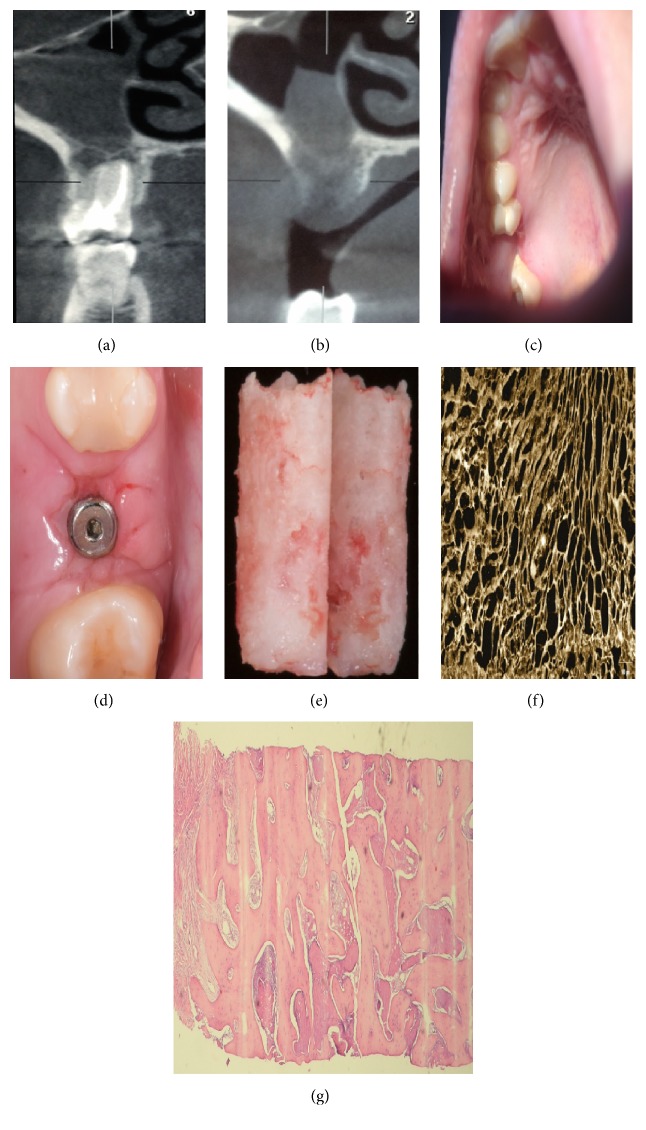
Patient of our service with odontogenic sinusitis. (a) CT scan before surgery. (b) CT scan 3 months after surgery. (c) Aspect of the mouth before the implant. (d) Implant site. (e) Biopsy of bone at the implant site. (f) Confocal microscopy of the bone recollected at the implant site. (g) Hematoxylin and eosin staining showing the presence of normal bone.

Due to the inability to perform biopsies of the surgical defect to confirm the presence of bone, the images of these patients were compared with those of a patient who developed odontogenic sinusitis requiring tooth extraction and functional endoscopic sinus surgery. The oral surgical defect was filled with L-PRF. Three months later, this patient had a cone beam CT scan preceding implantation revealing neoossification. In addition, the bone of implant site was sent for histological analysis, which confirmed the presence of normal trabecular bone formation by the hematoxylin-eosin staining and the confocal microscopy (Figure 3).

## 3. Results

5 of 44 patients undergoing endoscopic skull base surgery during this period met the inclusion criteria of having preoperative and postoperative CT. The cohort comprised four men and one woman, with an average age of 37.2 years and an average follow-up of 9.2 months (range: 4 to 24 months). Three patients presented CSF leaks and two had pituitary adenomas. CSF leaks were secondary to the resection of a plasmacytoma, blunt trauma, and a gunshot wound. One patient with a prolactinoma required surgery after stopping cabergoline in an effort to get pregnant; another patient presented with acromegaly (Table 1). All patients underwent reconstruction involving a nasoseptal flap without intraoperative or postoperative complications.

**Figure 4:**
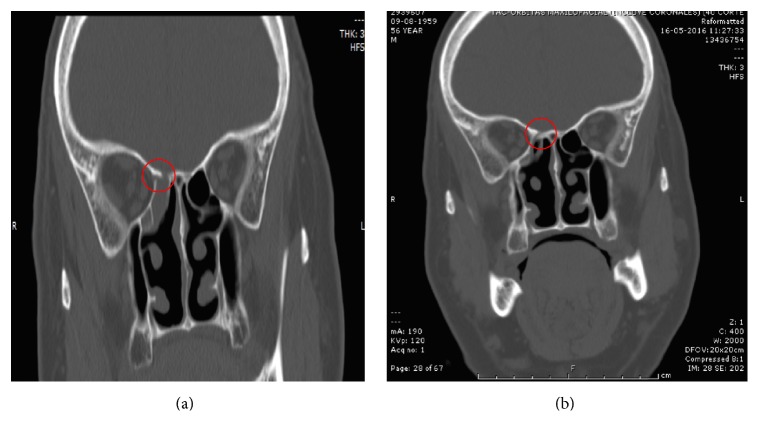
Case 1. (a) Preoperative CT scan showing a CSF leak in the right cribriform plate. (b) Seven-month postoperative CT showing a less dense bony closure of the surgical defect.

**Figure 5:**
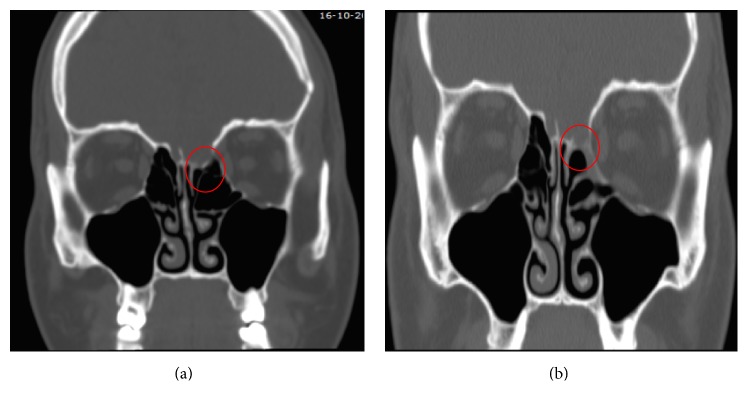
Case 2. (a) Preoperative CT scan showing a CSF leak in the left cribriform plate. (b) Seven-month postoperative CT showing a less dense bony closure of the surgical defect.

**Figure 6:**
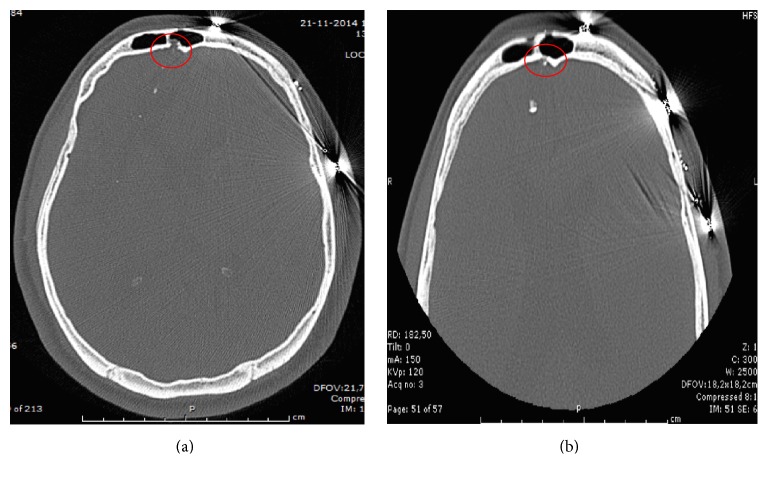
Case 3. (a) Preoperative CT scan showing a CSF leak in the posterior wall of the frontal sinus. (b) Seven-month postoperative CT showing a less dense bony closure of the surgical defect.

**Figure 7:**
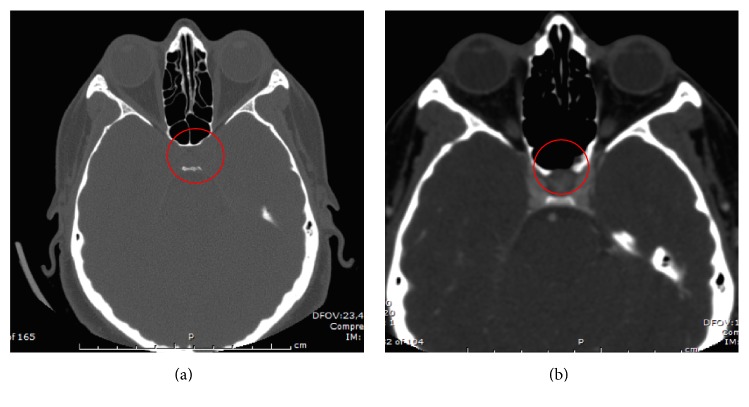
Case 4. (a) Preoperative CT scan showing a pituitary adenoma. (b) Seven-month postoperative CT showing a doubtful image of bony closure of the surgical defect.

**Figure 8:**
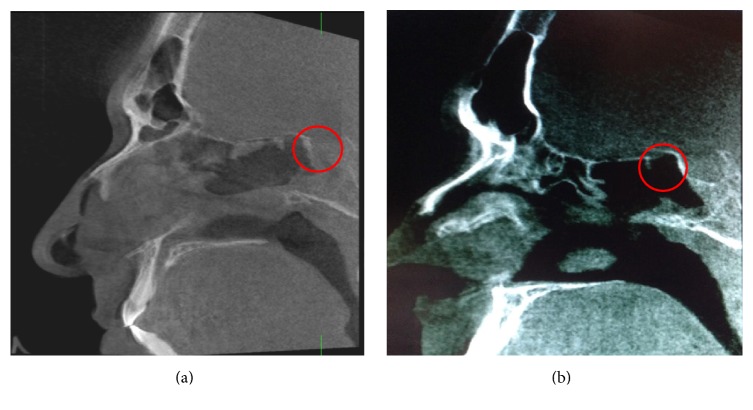
Case 5. (a) Preoperative CT scan showing a CSF leak in the anterior wall of the sella turcica. (b) Three-month postoperative CT showing a less dense bony closure of the surgical defect of the anterior wall of the sella turcica.

Postoperative CT images 3 months after surgery revealed a bony closure of the surgical defect, although the bone was less dense than the normal surrounding bone of the skull base. This imaging finding was seen in 4 of the 5 patients; in one case, the image was doubtful but clinically did not present complications or recurrence, without requiring revision surgery (Table 1). These images were similar to those of the patient with odontogenic sinusitis (Figures 4, 5, 6, 7, and 8).

## 4. Discussion

Leucocyte and platelet-rich fibrin (L-PRF) is a second-generation platelet concentrate widely used as an adjuvant for soft tissue healing and regeneration. Dohan Ehrenfest et al. demonstrated in vitro that L-PRF can stimulate the proliferation of mesenchymal stem cells and their differentiation into osteoblasts and that this effect was dose-dependent. Cell differentiation was measured by alkaline phosphatase activity and mineralization nodule formation [7]. Conversely, platelet-rich plasma stimulated cellular proliferation but inhibited cell differentiation [7]. Similar findings were obtained by Li et al. [6].

Bony closure may not be critical for most patients; however, there are several subsets of patients who may benefit from a rigid reconstruction like morbidly obese patients and patients with sleep apnea, postradiation edema, and brain invasion [9, 10].

Initial animal studies to evaluate the neoossification of surgical defects yielded disappointing results [11, 12]. Bölükbaşı et al. compared the efficacy of L-PRF versus biphasic calcium phosphate, a widely used product in dentistry as a filler, in the ossification of bone defects created in the tibia of sheep. Greater ossification was achieved using both products; however, L-PRF stimulated bone formation less than biphasic calcium phosphate alone, concluding that ossification induced by L-PRF depends on which adjuvant material is used [11]. A similar study by Knapen et al. comparing L-PRF with hydroxyapatite concluded that the only important factor in neoossification of surgical defects created in the skull of rabbits was time. No statistically significant difference was found when comparing both materials with the control group, alone or in combination [12].

However, recent studies on the role of L-PRF in neoossification have shown promising results [13, 14]. Li et al. demonstrated in vitro that L-PRF stimulates cell proliferation and differentiation of mesenchymal stem cells to osteoblasts by RunX2 gene. In vivo, it stimulates the formation of soft tissue in mice, closing bone defects by new bone formation in a span of 6 weeks. This effect seems more pronounced when using L-PRF in its lyophilized form, as it releases growth factors for a longer period [13]. Also, Wang et al. demonstrated in vivo that injecting mesenchymal stem cells plus L-PRF in the subcutaneous tissue of the back of mice is advantageous over injecting mesenchymal stem cells alone. Mesenchymal stem cells with L-PRF formed bone with a similar density to that of the spine bone when compared by computed tomography. Histological assessment of biopsies taken from the neoossification sites, compared with the normal spine bone, confirmed the imaging findings, suggesting that L-PRF is an important adjuvant to in vivo bone regeneration [14].

This pilot study yielded similar observations. Four of five patients showed closure of the skull base defect created during surgery as depicted by postoperative CT scans. Nevertheless, this new bone had a weaker bone enhancement as compared to the surrounding normal bone of the skull base. No postoperative complications were reported and no revision surgeries were performed. This preliminary data showed that L-PRF is an alternative to close surgical defects of the skull base created during surgery or repair CSF leaks and may induce the formation of new bone in the surgical site.

## 5. Conclusion

This pilot study suggests that L-PRF, used as an adjuvant in closing bone defects produced by anterior skull base surgery, may induce the regeneration of bone in the surgical site. However, more studies with larger numbers of patients are needed to confirm this exciting possibility.

## Figures and Tables

**Table 1:** Patients selected in this study. CT: computed tomography; CSF: cerebrospinal fluid.

Case	Diagnosis	Closure of surgical defect at 3-month CT	Revision surgery
1	CSF leak secondary to plasmacytoma resection	Yes	No
2	CSF leak secondary to trauma	Yes	No
3	CSF leak secondary to gunshot wound	Yes	No
4	Pituitary macroadenoma	Yes?	No
5	Pituitary macroadenoma	Yes	No
